# Framework for addressing occupational safety of de-sludging operators: A study in two Indian cities

**DOI:** 10.1016/j.jenvman.2021.112243

**Published:** 2021-07-01

**Authors:** Mamta Gautam, Kavita Wankhade, Gayathri Sarangan, Srinithi Sudhakar

**Affiliations:** a Independent Researcher/ Consultant, Kurukshetra, India; b Indian Institute for Human Settlements, IIHS Chennai: Floor 7A, Chaitanya Exotica, 24/51 Venkatnarayana Road, T Nagar, Chennai, 600 017, India

**Keywords:** Sanitation workers, De-sludging, Occupational safety, India, Fecal sludge management, Personal protective equipment

## Abstract

Safety of sanitation workers remains an often-ignored aspect in Fecal Sludge Management. While shifting workers from manual to mechanical means of de-sludging remains a priority, this paper highlights that there are a number of safety issues, including exposure to sludge, faced by sanitation workers, even in a mechanised context, where de-sludging trucks are utilised to provide de-sludging services. Based on a detailed analysis of observation of stakeholders and extensive process documentation (of de-sludging process), and expert interviews, the study identified three key safety concerns: inhalation of harmful gases, contact with sludge, and physical injury, and determined the underlying cause for the same, using a systems thinking approach. These causes are varied including behaviours and practices by households such as non-compliance of septic tank construction to design standards, irregular cleaning, improper disposal of inappropriate items in toilets; inappropriate or inadequate design of decanting stations, tools and equipment, and inadequate awareness and knowledge among all stakeholders. Using the hierarchy of controls framework, a set of measures are described to increase the safety of workers. These proposed interventions go beyond the provision of Personal Protective Equipment (PPE), and range from behaviour change campaigns, improvements in decanting stations, better access to appropriately designed tools. The study highlights the need to place emphasis on eliminating, substituting and controlling the hazards as necessary steps for PPE to be relevant. Finally, the paper places the issue of occupational safety within the larger context of the informal nature of de-sludging occupation and the overall vulnerability of workers. It posits that this makes safety more complicated to address as several factors need to be taken into account, and actions are required by multiple sets of actors.

## Highlights

• Safety concerns are prevalent even with mechanical desludging.
• Key concerns are exposure to harmful gases, skin infections and physical injury.
• hierarchy of control pyramid need to be addressed in totality.
• Measures to improve worker safety must address existing processes and practices.
• All stakeholders – households, workers, government are responsible to ensure safety.

## 1 Introduction

Fecal Sludge Management (FSM) is becoming a viable and accepted option for rapidly scaling up urban sanitation in India, either as a standalone solution, or as a complementary solution to networked systems.^1^ As implementation proceeds, there is a body of knowledge slowly building up based on practice. A critical yet often overlooked concern in FSM is the safety of de-sludging operators or sanitation workers who are responsible for de-sludging the Onsite Sanitation Systems (OSS) and transporting the sludge to appropriate treatment facilities. A recent study on health, safety and dignity of de-sludging workers highlighted a paucity of information on their challenges at work (World Bank et al., 2019). Workers are exposed to hazardous conditions while cleaning pits and septic tanks, often without adequate protective gear or equipment (MoUD, 2013).

Emptying of OSS and transporting the sludge in India is predominantly carried out by small private operators using a mix of manual and mechanical methods (MoUD, 2013, 2017), although some urban local bodies (ULBs) also offer this service. Manual cleaning, i.e without use of any mechanised aid like de-sludging trucks is both illegal and violates human rights. The primary legal instrument for ensuring the protection of sanitation workers is The Prohibition of Employment as Manual Scavengers and their Rehabilitation Act, 2013 (GoI., 2013a) which applies to all sanitation workers, including those cleaning septic tanks. Hazardous cleaning, that is any cleaning undertaken without the use of protective gear, cleaning devices and without following safety precautions, is banned. The Act further lays out penal consequences related to hazardous cleaning and places the onus of monitoring and enforcing these on a range of authorities, though enforcement remains patchy. The UN Special Rapporteur on the Human Right to Water and Sanitation states that basic human rights are violated when there is employment of manual scavengers (de Albuquerque, 2014).

The shift from manual to mechanical cleaning via suction trucks is the critical first step, and requires a range of enabling factors. Given that manual cleaning was widely practiced earlier, the transition to mechanised cleaning has not been uniform. For mechanised de-sludging, a wide range of vehicles are available from technologically advanced vehicles to small, retrofitted tempos. While mechanical de-sludging of OSS is increasingly becoming common in India, there are numerous safety concerns with the way it is currently practiced. This paper seeks to address these safety concerns in a mechanised context.

There is a paucity of literature related to de-sludging processes, practices and challenges faced by workers. Currently, a few government documents on septage management (MoUD, 2013; 2016, 2017), and standard operating procedures (CPHEEO, 2018) exist. Several newspaper reports on deaths related to the cleaning of septic tanks have been published. This paper seeks to add to the evidence on these practices in India and identify areas for improvement based on the study in two Indian cities. Specifically, this paper attempts to elaborate on the safety concerns at each step of the process of de-sludging based on experiences in the two cities. Informed on these by perspectives from sanitation workers as well as health and safety experts, suitable measures were identified and mapped on to the widely accepted hierarchy of controls, and are presented in this paper.

**Context**: The study was conducted in two Indian cities which have been anonymised to protect the identity of the sanitation workers and other stakeholders. In these two cities, de-sludging operators who offer cleaning services to households, establishments and industries, typically own one or two trucks with capacities ranging from 3000 to 8000 L. While households call when their tanks are almost full or if they are facing other problems, bulk generators such as commercial establishments and industries have a more regular arrangement. Requests for de-sludging are received over the phone although prices are negotiated after observing the site conditions. Sometimes, the driver is the truck owner himself. Each de-sludging trip includes a minimum of two persons – a driver, who also interfaces with clients to negotiate prices and a de-sludging worker who cleans the OSS.

**Organisation of the paper**: The next section presents study objectives, approach and methods. The following section presents an analysis of de-sludging process, and describes the emergent safety concerns. This is followed by a section on key findings from interviews with worker for their perspectives on safety. Based on the findings from the process documentation, interviews with workers, and analysis, a range of measures were identified along the Hierarchy of Controls which is presented next. This is followed by a discussion and conclusion sections. These sections depart slightly from the conventional norm of discussing only the research findings. Given that this research was conducted to inform programme design and implementation, these sections additionally reflect on the perceived challenges of implementing the safety agenda in Indian cities, and connect these to larger context of sanitation workers lives.

## 2 Objectives, approach and methods

The **objectives** of the study were to:

• analyse current de-sludging practices and identify resultant safety and health concerns for de-sludging workers;
• understand underlying reasons for these safety concerns/existing practices such as knowledge deficit and accepted behaviours;
• develop a set of preliminary recommendations to improve occupational safety of de-sludging workers; and
• understand the relevance and adequacy of legally mandated Personal Protective Equipment (PPE) as well as challenges faced by workers in using them.

### 2.1 Approach and methods

The team approached the study through a systems thinking lens, which meant that the issue of sanitation worker safety was approached holistically – the focus was not only on individual components, but also on understanding relationships between the components, and also understanding the underlying causal reasons for the persistence of safety concerns. This involved using a variety of methods from process documentation to participatory approaches and field testing kits. The methods evolved from the issues that the team observed on the ground; and the process was iterative rather than sequential – findings from one set of methods fed into another. In addition, techniques such as the ‘5 Why’ analysis was used to understand the underlying reasons for safety concerns, instead of stopping at most apparent ones.

Desk research, stakeholder interviews, de-sludging process documentation through observation and mock testing of safety gear kits were the methods adopted. The study was conducted in 2018 (April and May), and June 2019 in two cities. The findings from the first study done in 2018 were validated by the second study done in another city following a similar approach. While differences in the business models exist across the cities, the process of de-sludging is similar and are hence documented together.

**Desk Research**: A review of Occupational Safety and Health standards in India, USA and the UK was conducted. In addition, safety standards and protocols in other industries such as shipping, aerospace, construction and manufacturing industry, where safety protocols are observed, were studied.

**Stakeholder interviews**: Three sets of people – sanitation workers, owners who also worked as drivers, and drivers – were considered as primary stakeholders. Interviews were conducted with 26 primary stakeholders with an aim to understand their approach and perception to work, safety and health. Select interviews with owners of de-sludging services and a few others with clients were also conducted.

Fourteen experts in relevant fields such as safety, health, law and administration at the city and national level, were interviewed. The purpose of these interviews was twofold: (i) in the initial phase of the study to ascertain their perspectives on occupational safety to help frame the study, and (ii) to later validate the study findings.

**Process documentation**: The key method for identification of safety and health issues was observing and documenting the entire process of de-sludging – from receiving the order for emptying sludge to the final stage of disposal – in various settings, aided by video recordings. Each step was analysed in detail to understand the process itself, the risks posed by the settings as well as the prevalent practices, and the critical decision-making points.

The team took the necessary precautions to capture the de-sludging activity in an observational format, without directly intervening at any stage. This was done only after gaining the confidence of respondents through informal discussions. Observations of de-sludging of septic tanks were undertaken in the following settings across the two cities – households (26), factories (5), apartments (4), public toilets (4), hostels (2), college (1), bakery (1), hotel (1) and one site under construction.

**Testing sample safety kits**: As a first step, safety gears recommended in the Prohibition of Employment as Manual Scavengers and their Rehabilitation Rules,2013^2^ (GoI, 2013b) and gears used in shipping, petroleum and mining industries were screened and shortlisted. Upon further review, the safety auditor recommended gears used in the shipping industry for field testing as sludge from the shipping industry is closest to sludge in household and establishment settings. Based on the recommendations, worker preferences and market availability, safety gear sample kits were purchased to conduct a mock testing with the de-sludging workers.

### 2.2 Limitations

Caste plays a central role in sanitation work in India, and most de-sludging operations in the study cities and elsewhere are carried out by Dalits, a marginalised community. This has multiple implications including the ability of the workers to move away from the profession (due to stigma), and may perhaps also be a contributing reason for households continuing to insist on manual cleaning. While the study recognises the importance of caste, it was beyond its scope to address it.

In addition, the study included only male de-sludging workers, given that there were only a few women operators in the study cities. Many aspects of safety would have gender implications e.g., appropriateness of equipment both for ergonomic and cultural reasons, and social taboos that could potentially disable adoption of safety practices. This study has not been able to look at these gender implications but will do so in the next phase.

## 3 Key findings

The following sections highlight the findings from the process documentation of septic tank de-sludging in households and establishments, and emergent safety concerns.

### 3.1 Analysis of the de-sludging process

A complete de-sludging trip comprises the following steps: (i) receiving request from the client, (ii) reaching the site, (iii) emptying the OSS, and lastly (iv) disposing the sludge at an appropriate treatment site such as a decanting station/Sewage Treatment Plant (STP)/Fecal Sludge Treatment Plant (FSTP). The entire process takes anywhere between 40 and 70 min. Factors such as the availability of removable septic tank lids/manholes, time taken to allow harmful gases to escape, distance between the site and decanting station, and traffic on the road influence the total time taken. The exact location of the septic tank in the household also has a bearing on this as the greater the distance from the main road where the vehicle is parked, the more difficult and time-consuming the process, as several suction hoses need to be connected.

**Fig. 1:**
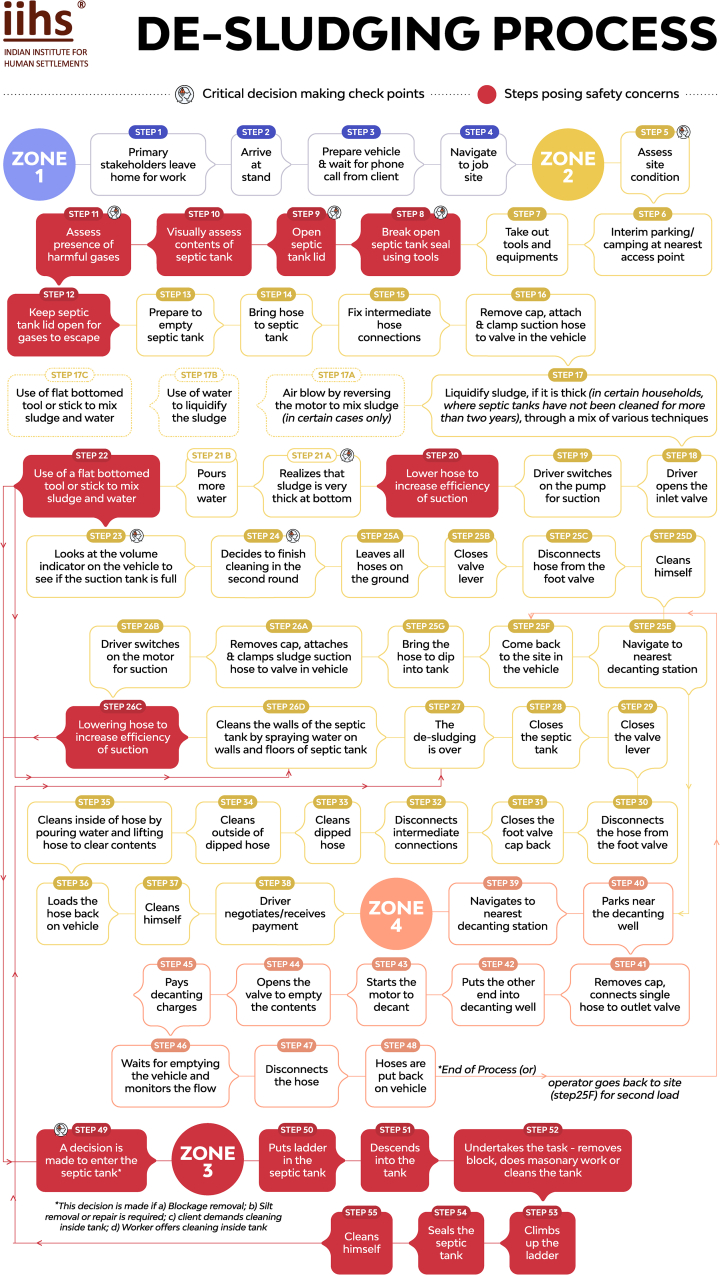
De-sludging Process.

The entire de-sludging process can be broken into a sequence of 55 steps across 4 Zones, as illustrated in Fig. 1. Zone 1 is the journey to the emptying site, Zone 2 is the actual process of de-sludging, and Zone 4 is disposal at final site. Zone 3 is not applicable to all operations as it only pertains to instances where workers enter the OSS either for functional or behavioural reasons. As is evident from Fig. 1, most of the critical decision points exist in Zones 2 and 3. A brief description of the processes in each of these zones is provided below along with associated safety concerns.

***Zone 1****:* Workers reach the vehicle parking area, where they clean their vehicles and wait for job orders for the day over the phone. Once they receive a job order, they set out immediately to the client site.

***Zone 2****:* Upon reaching the site, the de-sludging vehicle is parked at a point closest to the septic tank, taking into consideration access and road width.

**Site Assessment**: The first step is to conduct a site assessment to gauge the distance between the septic tank and vehicle and to decide on the number of suction hoses required. The location of septic tanks in houses could be in front, on the side, or behind the house, and in some cases below the super structure of the toilet. Reaching and accessing the tank often poses a risk of bruises, wounds and injury especially to feet, knees and thighs, as workers navigate around thorns and bushes with slippers. These injuries may be minor, but if left unattended, can become infected leading to other health problems.

**Opening the lid**: The next step is to decide how to open the lid or slab, based on the type and condition of the septic tank, especially the covering. If the septic tank has a removable lid, workers are able to set it aside with their bare hands. However, several septic tanks are completely sealed structures, whose tops need to be broken to gain access to the tank. Workers rely on experience to gauge the structural quality and strength of the septic tank for further action. A crowbar and hammer are used to break or remove the entire rectangular or square slab. Lids are too heavy to lift with bare hands causing injury to the worker's extremities. It was also reported that in the process of opening the lid, gases are sometimes inhaled, resulting in dehydration and nausea in some cases. Sometimes, chemical reactions within the OSS corrode the lid, and cause the slab to collapse. This poses the danger of the worker accidentally falling into the septic tank, exposing them to physical injury, harmful gases and fecal matter.

**Ascertaining sludge characteristics**: Once the septic tank lid is opened, the nature and viscosity of the sludge is checked by bending down which can lead to a fall or exposure to harmful gases.

**Checking for harmful gases:** The next check detects the presence of harmful gases in the septic tank by checking the vent pipe. A well-placed air vent indicates that a septic tank may not have harmful gases trapped inside. If the vent pipe of a septic tank is absent, not correctly installed or malfunctioning, harmful gases could accumulate inside the tank, which could be inhaled by the worker causing a loss of consciousness or, in some instances, death. Workers also experience physical symptoms in the eyes (irritation/burning/watering), and nose (breathlessness and pungent smell). In the absence of gas detection equipment, workers take these symptoms as an indication of the presence of gases. The presence of cockroaches is considered as a sign of oxygen being present and their absence, therefore, is perceived as a concern. Some novice workers also use a flame test to check for gases, which further exposes them to the risk of skin burns. If harmful gases are detected, they wait for them to escape, before commencing operations.

**Connecting the hose**: Attaching together intermediate suction hoses to establish a connection between the vehicle and septic tank, removing the inlet and outlet valve cap and putting them back, are all done with bare hands. In fact, connecting the last hose to the vehicle is done by one person by placing the hose between the thighs for the sake of better grip, thereby increasing the risk of contact with fecal sludge. Workers are also susceptible to physical injury while working with such pipes as well as coming into contact with objects (likely to have rough edges) on the ground.

**Desludging**: Once ready, the inlet valve in the truck is opened, vehicle motor for suction is switched on, and the tank is emptied by lowering the hose inside. This is done slowly to remove the sludge without any blockage and to ensure that only the bottom part of the hose touches the sludge. If the sludge is found to be too thick for suction, tools are used to mix the sludge – blowing air by reversing the motor (only in some cases), mixing water with thick sludge, and mixing or scraping sludge with a stick or flat-bottomed tool. To lower the hose or mix sludge using tools, workers bend down from their waist to get close to the tank which could lead to an accidental fall, especially if the worker is not experienced.

Upon completion, the client inspects the tank, and if the job is satisfactory, the septic tank is closed. In some cases, it is completely sealed with cement and sand. Hoses are disconnected and tied back to the vehicle after cleaning, and the truck proceeds to the decanting station or disposal site, which falls under Zone 4 activities.

***Zone 3***: Entry into septic tanks is unlawful, especially if undertaken without adequate safety precautions. Entry happens due to two reasons:

(i) Functional reasons such as blockage removal (if it cannot be done from outside), undertaking repair and maintenance of the tank, or silt removal, and
(ii) Behavioural/societal norms where the households ask the de-sludging workers to clean the tank from inside or workers themselves offer to do so for an additional fee.

Workers enter the tank with the help of a ladder, manoeuvre through thick sludge and inspect the tank with a torch to determine the course of action. Once the block is identified, it is removed using a stick or a piece of cloth and, with the help of the driver, brought outside. Subsequently, the hose is used to suck out the sludge. There is a lot of coordination and communication between the worker inside the septic tank and the driver outside the septic tank. Once the tank is clean, the worker cleans himself with water, comes out, and closes the tank.

Entering septic tanks is extremely dangerous and potentially fatal. Just the act of entering the tanks using the ladder may cause injury in case of an accidental fall. These risks are particularly pronounced as workers wear minimal clothing and do not use safety gear. Without adequate protective gear, the entire body (except face) is exposed to fecal sludge. Inhalation of harmful gases and/or asphyxiation while in the tank can cause unconsciousness or death. Further, the presence of multiple objects blocking the tank including menstrual products, condoms and polythene bags increases time to complete cleaning, thereby increasing the duration of exposure.

The workers felt that PPE was necessary for entry into septic tanks and requested for the same. Given that it is unlawful as well as a violation of human rights, this study does not seek to make this step ‘safer’ but examines the underlying reasons for entry into the septic tanks, and how to eliminate the same.

***Zone 4****:* Upon parking the vehicle at the disposal site, the worker connects the suction hose to the outlet valve in the truck and the rear end of the pipe is placed in the decanting well. The vehicle motor is turned on to empty the sludge into the well, while the worker and driver take a break.

Some steps might be repeated as the desludging operator may make multiple trips depending upon the size of the septic tank, volume capacity of the vehicle, and sometimes, for the opportunity to make more money.

### 3.2 Key safety concerns

Safety concerns identified through process observation and stakeholder interactions were further elaborated by experts. The key safety concerns identified for de-sludging workers were exposure to harmful gases; direct contact with sludge; physical injury. In addition, skin burns were also a safety concern.^3^^,^ 4

**Inhalation of gases:** A combination of factors such as sealed tanks (no vent pipe), long de-sludging cycles, and use of chemicals for toilet cleaning lead to the accumulation of gases in the OSS. Studies have shown that ‘the concentration of gases varies based on factors such as the frequency of de-sludging intervals, sludge composition, temperature and pH’ (Hariharan et al., 2016). While a more thorough testing and evaluation is necessary, several newspaper reports seem to indicate that inhalation of gases could cause death – either directly or indirectly (for examples of these newspaper reports, see Indiatimes^5^ and NewsClick^6^). These reports highlight that inhalation of gases have led to unconsciousness, thereby causing a worker to slip. These falls have resulted in death either most probably due to fatal injuries or asphyxiation. While the actual cause of death warrants a thorough study, there is enough evidence to show that inhalation of gas is a critical safety concern.

While there is limited evidence on long term health consequences, discussions with health experts indicate that prolonged and passive breathing of harmful gases can cause breathlessness, asthma, and other lung and respiratory diseases. All the experts interviewed were unanimous about workers suffering from bronchitis and other breathing-related problems due to inhalation of gases. At present, workers are unaware of safe and alternative methods such as gas monitors to test for harmful gases. Even with awareness, there are likely to be other concerns like lack of affordability of these monitors, and difficulty in usage.

**Direct contact with sludge leading to skin infections:** Workers are at risk of direct contact with or splashes of fecal sludge at multiple stages in the de-sludging process which is aggravated as workers seldom wear proper PPE. While there is limited evidence on the impact of contact with sludge, studies on sewage workers reveal conditions such as leptospirosis, hepatitis, dermatitis and helicobacter pylori infection (Tiwari, 2008). It is highly likely that de-sludging operators also suffer from similar conditions, given the similar nature of fecal sludge and sewage (though the exact characteristics differ).

In addition, disposal of household products such as bathroom cleaners, shampoos, and detergents containing chemicals in the OSS seems to aggravate skin-related diseases. Sanitation workers who have been in the profession for decades report a rise in skin infections with the increased use of household chemicals. Many workers often complain of skin infections like itching and white spots, while doctors report treating them for eczema and psoriasis. Experts in the study also report gastrointestinal problems like diarrhoea, parasitic infections and musculoskeletal problems amongst workers.

**Physical injury:** Breaking open the septic tank lid and lowering of the hose pipe are potential sources of physical injury. Risk of physical injury is pronounced inside the tank as invisible sharp objects such as glass pieces, spoons and razors in the sludge, cause injuries to the workers’ extremities, since many do not wear protective gear and clean with bare hands. Additionally, if septic tanks are placed in difficult-to-access locations, workers are exposed to insects, thorny bushes, and overflowing or stagnant water.

## 4 Worker perspective on safety, including use of protective equipment

This section summarises findings from interviews with workers on their perception of safety and risks, and their current coping mechanisms and practices.

**Fig. 2:**
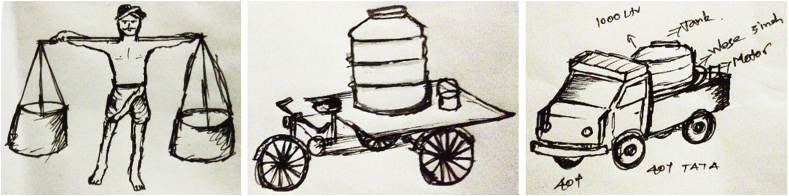
A Worker's Illustration- Journey of De-sludging.

Workers have incrementally changed their practices to improve efficiency of emptying and transportation, while minimising direct contact with fecal sludge. Earlier, fecal sludge from OSS was emptied manually using buckets. At the next stage, emptying was still done manually but filled in larger tanks and transported by carts. Subsequently, older trucks were retrofitted with tanks and suction equipment to enable both emptying and transportation. Now, in the two study cities, purpose-built suction trucks with varying capacities are under operation. Fig 2 depicts an illustration by a sanitation worker of his journey.

Workers feel that the mechanisation of de-sludging operations has limited their exposure to sludge and enhanced occupational safety. Hence, the relatively minimal exposure to sludge while handling hoses is not perceived as a major concern. Further, few workers believe that their longevity in this profession has helped them develop a certain degree of immunity, and hence they undertake de-sludging without using safety gear.

In general, workers are aware of the law prohibiting manual scavenging and manual entry into OSS. While some workers have not reported entering a septic tank keeping them in compliance with the law against manual scavenging, others have reported instances of entering septic tanks. One of the key reasons remains blockages, for which workers call for responsible action by clients.

‘Clients need to learn that the septic tank is only meant for fecal sludge and not for foreign objects/sharps or menstrual products. We have cut our hands so many times while cleaning the pipes or tanks. We face injuries because of client's actions. Yet, we clean everything that you make a mess of.’ – A worker.

Key design problems highlighted by workers include the absence of vent pipes, difficult to access septic tanks, and lack of opening to insert hose pipes. They also report poor slab characteristics – being either too heavy to lift or too light to take the load as a concern and prefer a two-compartment septic tank which allows for separation of solids from water.

Workers have their indigenous methods of coping with hazards. They deal with harmful gases by checking the vent or pipe, or by observing their own physical symptoms. Some novice workers also do flame test by lighting a matchstick, paper or lamp. If they burn, the inference is that gases are present, in which case they wait for them to escape before starting to de-sludge. Coconut oil is used to coat their skin, as a method to restrict contact with sludge. Further, washing extremities with soap after contact with sludge is also practiced. Physical injury is dealt with immediately by applying mud which, is believed to be a temporary first aid, while proper medical help is sought later. They cope with the odour by covering their face with a towel. They also report consuming alcohol and tobacco to cope with work related stress.

With the use of PPE made mandatory by law, the two ULBs in the study cities have sensitised workers on the need for safety gear. Owners of de-sludging vehicles have provided basic items such as gloves and boots. However, in reality, PPE is seldom used. Workers are aware of the importance of such gear and expressed specific need for gloves and masks but cite many constraints in using currently available equipment. For instance, the gloves fit poorly, cause sweating and boils, reduce speed, and hinder work while lifting heavy objects. Boots have poor grip besides continuing to allow sludge to enter them while inside the tank. Currently available surgical masks are unusable after the first use because of smell, sweat and possibility of infection due to re-use.

Besides design issues, other concerns including poor quality, recurring maintenance costs, and storage of used PPE reduce the value proposition of using PPE. Given that the de-sludging activity lasts for about 45 min and the risk is not continuous through the day, exposure to sludge is not perceived as a major concern. Owners of de-sludging vehicles also complain that workers do not wear PPE despite its provision due to the reasons noted above. They felt that better-quality safety gear could potentially have a bearing on usage.

Participatory exercises were conducted to understand workers’ preference for PPE. These revealed a preference for safety gears in the following order: gloves, respiratory mask, gas monitor and gumboots, goggles, helmets, jackets and safety cones. Further, interviews were also conducted to understand preferences for specifications of each individual equipment. Workers preferred weather- and spill-proof, arm length gloves with good grip and fit. They also favoured easy-to-clean gloves, and waterproof gumboots with good grip across all surfaces and protection from sludge and thorns. Snug fit, waterproof and communication-friendly masks which offered protection from harmful gases and sludge were preferred. They also sought masks attached with goggles for ease of use.

The most commonly reported occupational health problems were skin issues, cuts, bruises and wounds. They do acknowledge that limited access to medical facilities meant that chronic conditions remain undiagnosed. Importantly, although workers complain of health issues, they are unable to link these complaints with occupational safety as they do not understand the causal connection.

## 5 Emerging framework to address safety issues for sanitation workers

The three concerns – exposure to harmful gases, direct contact with fecal sludge, and physical injury – can be addressed, and the de-sludging operations made safer only through enabling wide ranging measures (varying from behaviour change communication to the use of PPE), and involving a range of stakeholders. These measures were identified through analysis of de-sludging operations, discussions with workers and experts, interviews with select manufacturers and supplies (of trucks, tools and equipment) and limited field testing.

**Fig. 3:**
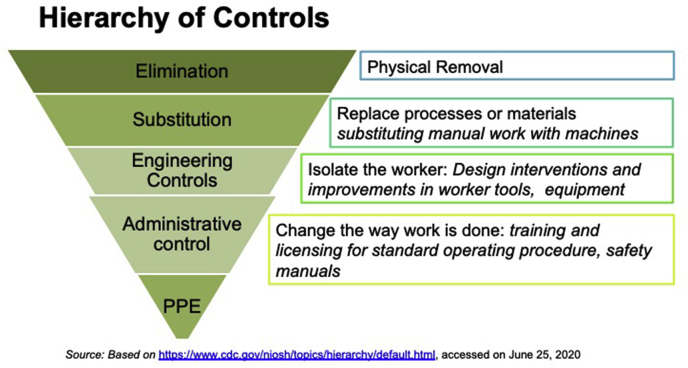
Hierarchy of Controls.

These shortlisted interventions were then mapped onto the widely accepted Hierarchy of Controls, which is identified by an inverted pyramid and comprises five parts – elimination, substitution, engineering controls, administrative controls and PPE (Fig. 3 Hierarchy of Controls). The measures along this pyramid with definition of each of the steps referred from CDC are presented in the section below (CDC, n.d.).

### 5.1 Elimination

Elimination is physically removing the hazard. It calls for removal of work, which, in this case, is the removal of de-sludging itself which cannot be eliminated. However, entry into the OSS can and should be eliminated in most cases. This is in line with The Prohibition of Employment as Manual Scavengers and their Rehabilitation Act, 2013. As stated earlier, the reasons for entry could be due to functional or non-functional reasons. The most common ‘functional’ reasons for entry are blockages and thickening of sludge due to infrequent cleaning. Several functional reasons can be addressed through design interventions or behaviour change campaign for both workers and clients.

Non-functional reasons, like misunderstanding that OSS need to be completely cleaned, however, are more difficult to address, but a start could be made by educating clients and workers on the illegality of manual scavenging and its penal consequences.

Awareness interventions could include the following:

• Masons and clients: on proper construction of septic tanks as per standards, with regard to vent pipes and removable lids
• Clients: on not disposing non-degradable waste and other objects which block the inlet to the septic tank necessitating entry for cleaning; on the importance or good practice of leaving behind some sludge while cleaning as ‘seed material’ for digestion of subsequent inflows, and that a full cleaning may be counterproductive.
• Workers: on cordoning off unattended open septic tanks, and on the importance or good practice of leaving behind some sludge while cleaning as ‘seed material’ for digestion of subsequent inflows.

### 5.2 Substitution

Substitution is replacing a material or process with another that is considered to be less hazardous. In terms of the de-sludging operation itself, the primary substitution is to replace manual cleaning methods with mechanical methods. While this is not relevant to the study cities, manual methods of cleaning exist in many cities, and require substitution by mechanical methods such as trucks, or even by other equipment such as gulpers. In instances where de-sludging trucks are already being used, a possible substitution mechanism is to replace the chemical products used for cleaning toilets with non-chemical ones.

### 5.3 Engineering controls

Engineering controls are used to isolate the worker from the hazard. ‘Well-designed engineering controls are highly effective in protecting workers and will typically be independent of worker interactions’ (CDC, n.d.). Potential engineering solutions which need to be explored include:

#### 5.3.1 Blockage removing tools

Removal of blockage has been identified as a key challenge by the workers and is a main reason for entering the OSS. Given this, two key tools are necessary to:

• Liquify sludge through tools or a water pressure mechanism; and
• Remove stubborn blockages using the principle of force or force and drilling.

A market availability and efficacy study needs to be conducted to examine currently available tools used for de-sludging or in other industries/operations.

#### 5.3.2 Air blowing technique to reduce exposure to gases

Both the vehicle and the suction hose can be a cost-effective means to eliminate gases in the tank by using the air blowing technique. It simply involves blowing air into the tank after it is opened, so that all gases are released. It also reduces waiting time (by 15–30 min) for workers. Some de-sludging vehicles already have this feature. While this technique has been reported as being used in large septic tanks, its efficacy must be tested for smaller septic tanks in households. If effective and successful, this could be included in the standard operating procedures.

#### 5.3.3 Design and construction of septic tank

Despite existing standards for construction of septic tanks, they are seldom built as per norms as practising masons are not trained on such standards. Owners of houses, where these OSS are predominantly built, are also unaware of the same. Absence of compliance to prescribed norms poses a threat to safety. Two key aspects are to be kept in mind:

*Vent pipe:* The absence of vent pipes leads to the accumulation of gases, with undesirable consequences. Also, where available they do not always meet standards. Vent pipes need to be constructed and placed 2 m (BIS: 2470-1 (1985)) above the top of the building to prevent inhalation of harmful gases.

*Improvements in lids:* This study indicates that safety can be enhanced by improving the design of lids. The characteristics already included in the standards are: removable lids that need not be broken every time, which are structurally strong for anticipated loads. In addition, lids should contain space for two hoses and allow for air-blowing through the suction hose in the septic tank. Other innovations such as septic tank riser/netting/grating for those septic tanks which are buried in the ground could be considered.

Two other design considerations for improving safety are to build a septic tank with appropriate slope to aid de-sludging and to build an inspection chamber in the same place as the inlet valve, which will allow both visual and physical access to the worker to address blockage, rather than having to enter the tank.

#### 5.3.4 Improvements in vehicle design

Loose inlet valves and clamps could possibly lead to sludge spillage while impeding suction efficiency. The key recommended steps are:

• Regular checks and preventive maintenance of the vehicle (e.g., checking for wear and tear)
• Design improvements in vehicle spout, clamp, handle of trucks/dip pipe to minimise contact with sludge. It is also necessary to explore possibilities for storage of the de-sludging equipment.

#### 5.3.5 Improvements at decanting station/STPs/FSTPs

Improvements need to be undertaken at the point of disposal whether these are FSTPs, or pumping stations or STPs (in case of co-treatment). The angular slant on the ground at the disposal point must be verified so that the vehicle can empty completely without much use of the suction motor.

Another urgent requirement, which has also been identified as a need by the sanitation workers, is to provide access to adequate sanitation and washing facilities at treatment plants. It is ideal to equip the plants with first aid kits and provide basic medical facilities, including organising periodic visits by a doctor.

### 5.4 Administrative controls

They seek to limit the exposure to hazards rather than remove it. ‘Administrative controls are relevant when hazards are not well controlled’ (CDC, n.d.), and hence have relevance for de-sludging operations.

The study reviewed the existing codes and standards, which fall under three primary categories: occupational safety for sewerage systems such as CPHEEO 2013; BIS: 11972 (1987); generic safety practices such as BIS:18001 (2007) and OSHA 3151-12R (2004); and guidelines for septic tanks BIS 2470-2 (1985) and ASTM C1227-20.^7^ Available literature can aid in the development of a safe operating procedure for de-sludging operations. However, de-sludging operations as a separate area of work has been gaining traction only in recent times and the nuances of such operations involving a human element require critical attention. These have not been completely addressed in the ambit of existing laws and codes of practice.

This study identifies the following steps for action:

**Standard Operating Procedures (SOP)**: It is important to draw up an SOP which will offer step-by-step instructions to carry out complex, routine operations in a correct and consistent manner. In the context of de-sludging operations, a detailed SOP should cover the following:

• Wearing and removing of safety gear,
• Assessing harmful gases and precautions so they are not inhaled, and
• Removing blockages while averting any kind of hazard.

The SOP needs to consider the key risks and safety concerns at different stages of de-sludging to ensure total safety. In addition, it is important to frame emergency protocols in the event of accidents during de-sludging operations.

Post the completion of this study, in 2018, the Ministry of Housing and Urban Affairs, Government of India, issued an SOP for mechanised cleaning of septic tanks and sewers (CPHEEO, 2018). The SOP addresses de-sludging as a separate area of work and provides the basic process/methods for de-sludging, emergency preparedness and list of PPE to be used during operations. Implementation of SOP on the ground, and resultant impacts will need to be determined in the future.

**Improved Enforcement, Monitoring, and Awareness**: Deviance from design standards for OSS increases risks. Therefore, ensuring that new OSS constructions follow the requisite standards, as well as ensuring that older OSS are retrofitted could mitigate these risks. Periodic monitoring by the ULBs is necessary to confirm that protocols are being followed. In addition, workers need to be sensitised on relevant laws, safety standards and protocols.

### 5.5 Personal Protective Equipment

While considering worker safety, considerable efforts have been directed towards provisioning of PPE. While this is a step in the right direction, one needs to remember that PPE does not eliminate hazards; there is a danger of workers being exposed if equipment fails. Therefore, PPE must remain the last line of defence.

The focus on PPE has been around provisioning alone, rather than also addressing issues of design, use and training which are critical. PPE needs to be appropriately designed, and suitable for specific contexts, failing which it can increase the risks it is meant to protect against. Inappropriate design is one of the reasons for workers not using PPE, the other reasons being not perceiving the risks, and not knowing the long-term health consequences of hazards which PPE could potentially help mitigate.

The study findings indicate a two-pronged strategy for addressing the above concerns. One, it is hoped that providing appropriate and well-designed safety gear will address functional reasons around non-usage of PPE. This needs to be supplemented by training on usage of safety kits. To address non-functional reasons, appropriate training and awareness programmes must be conducted on the importance of PPE.

**Fig. 4:**
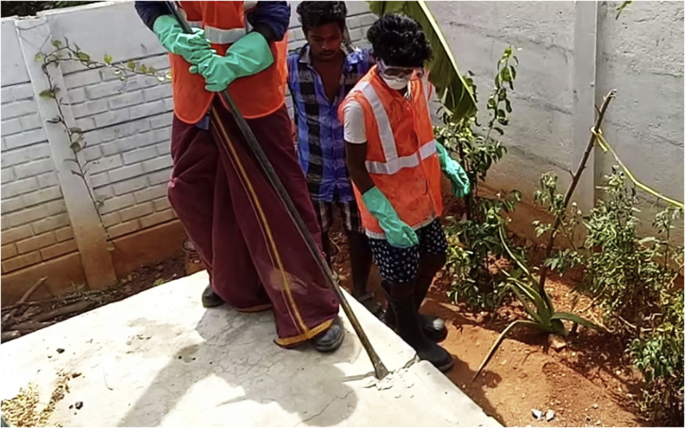
Images of field testing of the PPE- Opening a septic tank.

**Fig. 5:**
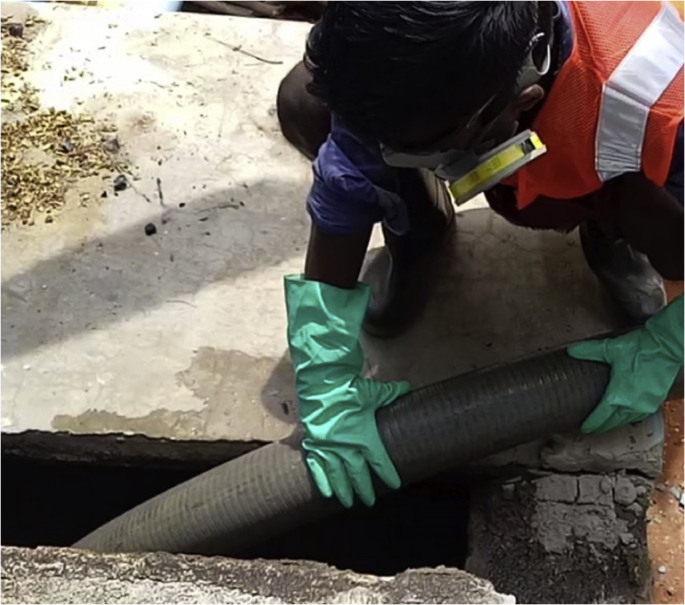
Images of field testing of the PPE - Inserting a hose pipe.

Testing of safety kit was conducted to get insights into designing and adaptation of PPE. Two sets of field tests were done. The first tested six types of gloves available in the market since gloves were the most preferred safety gear by the workers. The second tested a complete sample kit in actual field conditions, albeit with limited brand options.Fig. 4, Fig. 5 show images of field testing. In addition to gathering feedback from the workers, their intuitive responses to functionality of the gear were observed

Based on the legally mandated equipments, analysis of safety gears used in other industries, worker preferences (through participatory exercise mentioned above and field observations), a sample safety kit was shortlisted by an expert which included the following: gloves, masks (face mask and breathe (particulate) mask), gumboots, goggles, gas monitor, reflective jackets, caution tape and safety cones. Further, for each item in the safety kit, specific brands/designs were selected based on worker feedback on ideal/preferred features and market availability.

Key findings from the testing of the sample kit are presented below.

**Gloves**: Nitrile gloves were preferred over cotton, wool and plastic. Elbow length gloves offered better protection while de-sludging and customised sizes were very important for good grip. One pair of gloves for each worker per operation with spares was needed so that they could be washed and dried (three per day).

**Gumboots**: These offered better protection against thorns and shrubs, provided the fit was right. However, they hindered climbing into the vehicle and did not protect against water entering while washing pipes and spillage. Gumboots were preferred in the rainy season, while for other times, open footwear which was easy to dry, was preferred.

**Face mask:** Air purifier mask for the person near the septic tank, and breathe/particulate mask for the person away from the septic tank were tested. Both masks were found suitable for use while de-sludging in household settings although training on use and maintenance was required. Further studies are required to check suitability of use in commercial places.

**Goggles**: Two models of goggles – one which can be worn with an air purifier mask and the other without it were tested. Both were found suitable for use, although training was required on use and maintenance.

**Gas monitors**: They were found important for use although further testing and training with workers is required as the interface of the equipment is complex.

The findings from testing sample safety kits highlight the need to adapt/redesign PPE to address the specific requirements of de-sludging workers in Indian conditions, including easy maintenance. As a way forward, there is a need to:

• Adapt or redesign existing gear – especially gloves and boots;
• Evaluate suitability of respiratory masks already being used in other allied industries.

This testing was an open-ended, exploratory study to understand safety aspects, develop an emergent framework, and enlist some innovative ideas, not recognised earlier, in the context of worker safety. In continuation of this study, the research and field team is checking feasibility of each of the interventions proposed in earlier sections through further research, on-site testing – working in close collaboration with all stakeholders. Depending on the resources, capacities and finances, select measures will be implemented and scaled up.

## 6 Discussion

Till recently, much of the focus on worker safety has remained on PPE. While PPE is essential, it is important to reiterate the PPE is the last line of defence, and a range of steps can and should be taken prior to this stage to ensure worker safety. Focus on worker protection through PPE without steps to eliminate, substitute and control hazards is unlikely to yield desired results. By taking a systems thinking approach, this research has highlighted a number of entry points that are not conventionally associated with sanitation worker safety, such as to change behaviours of households.

Further, occupational safety needs to be viewed within the context of the informal nature of the sector. This includes informal nature of de-sludging practice, but also those of masonry and construction practices, and manufacturing of trucks.^8^ This has multiple implications, first one being the wide variations in containment systems, design of trucks and sometimes even safety equipment.^9^ Such variations could be within an urban area but will certainly vary across urban India's vast geographic and economic landscape.

This brings into question the feasibility and salience of a generic set of guidelines across large swathes of cities. The implication is not that each city should develop a unique set of guidelines, but that some amount of customisation may be critical and essential. This is applicable not only to protocols, but also to the development of tools and other initiatives. It is worth reiterating that the workers themselves are best placed to understand these differences and help ideate customised solutions which can be adapted to local needs.

The informal nature of operations also brings up questions of feasibility of implementation and scaling. The de-sludging operators, who have often risen from being manual scavengers themselves, run relatively small enterprises, either functioning as a driver, or employing a few workers – even though there could be some exceptions to the scale of operations. It may not be feasible for these small enterprises to purchase the necessary equipment or tools. More importantly, these are not one-time purchases but involve recurring maintenance expenses.

It is also not clear whether ULBs, with their stretched finances will be able to meet the costs for the above requirements, or indeed even consider it their mandate. As has been demonstrated during the Covid-19 pandemic, sanitation workers on payroll or contracted directly by the ULBs were provided PPE, but informal, private workers like the de-sludging operators were often left out. Some of the interventions require households to invest money in retrofitting, which again remains a difficult ask.

Given that this research was conducted as a prelude to inform practices on the ground, a small note on the methods is not remiss. Instead of examining the feasibility of pre-determined solutions, or even investigating only known safety concerns, the starting point was intense observation and documentation of the de-sludging process itself. Further methods like testing or even selection of experts was done, based on what was discovered on the field. This is not to undermine the importance and necessity of learning from existing literature and practices, but to place experiential knowledge – including and especially that of workers – at the centre. The study also reinforces the necessity of multiple methods, drawn from several disciplines and professions. While this remains the subject matter of another paper, the importance of an interdisciplinary team – designers, sociologists, community workers and policy experts – working together needs to be highlighted not only for the insights that were brought to the table, but also for what was learned through the conflicts (in approaching the problem as well as the measures) among the team.

## 7 Conclusion

Occupational safety needs to be viewed not only within the informal nature of the sector, but also within the precarity of the worker's life. There is no doubt that no safety initiative will be successful without the sanitation workers taking responsibility, but this needs to be seen within larger contexts they operate in. One, this research shows that a range of stakeholders are responsible for safety. For example, local practices and household considerations of cost and space have taken precedence over septic tank construction norms, which has resulted in non-standard containment structures that are often more hazardous to clean. These practices need to change. *Responsibility for safety lies with multiple stakeholders.*

Second, one of the current narratives is that sanitation workers are negligent about their own safety. *This needs to be countered.* An example of such a narrative is that sanitation workers are given PPE, but that they do not want to use it. Our interactions with sanitation workers show that there are often valid reasons for not using the PPE given to them, including the probability of the gear increasing their risk and exposure to danger if the gear is inappropriately designed and selected. For instance, gumboots that do not offer good grip could actually increase the risk of injury, and sometimes there are reports of gloves causing boils and blisters or hindering lifting of heavy items.

Third, *sanitation workers must be included in the process not as beneficiaries but as active participants.* During the course of field work, it became clear that given their long decades of experience of working in this business, sanitation workers have immense amount of tacit knowledge – both about the risks as well as indigenous methods to improve their safety. Applying this knowledge in innovations in the de-sludging process will be valuable. Any set of measures should not be foisted upon them.

Finally, *occupational safety needs to be viewed within the larger context of the sanitation workers' life and work*. While accusations are made against sanitation workers being negligent or unaware about their safety, it has to be noted that in the absence of adequate social protection nets, their dominant ‘safety’ and ‘health' concerns may not be occupational safety ones. Rather, the lack of access to medical facilities for their families, unsafe home conditions, and stigma and hostility from society, are some concerns (that have safety implications) that could take precedence in their minds. While this paper does not address these broader concerns, the information gathered over the course of fieldwork emphasises that occupational safety cannot be examined in isolation and must encompass the broader well-being of the workers and their empowerment.

## Note

This paper is based on a detailed report titled ‘De-sludging Operators: An assessment of occupational safety in two Indian cities’.

## Credit author statement

**Mamta Gautam:** Conceptualization, Methodology, Investigation, Project administration, Validation, Formal Analysis **Kavita Wankhade:** Conceptualization, Supervision, Formal Analysis, Writing - Original Draft, Writing - Review & Editing. **Gayathri Sarangan:** Writing - Original Draft, Writing - Review & Editing**. Srinithi Sudhakar:** Project administration, Writing - Original Draft

## Declaration of competing interest

The authors declare that they have no known competing financial interests or personal relationships that could have appeared to influence the work reported in this paper.

## Footnotes

1 OSS are the predominant sanitation arrangement for urban households in India, with about 45.3 per cent connected to septic tanks and pit latrines, and 32.7 per cent connected to sewers (Census, 2011). Therefore, proper and adequate FSM systems are essential for achieving total urban sanitation in India.

2 The Prohibition of Employment as Manual Scavengers and their Rehabilitation Rules, 2013 lists 40 kinds of safety gear and 14 cleaning devices to ensure safety.

3 Interviews with experts and workers also identify alcohol consumption as a key health concern, but it is beyond the scope of this paper to address this issue.

4 Skin burns occur when worker tests for presence of gases by lighting a lamp, candle or paper, thus setting fire to potential combustible gases in the tank, resulting in burn injuries.

5 Tamil Nadu: House owner, his two sons among six dead after inhaling toxic gas from septic tank (indiatimes.com)India Times, 2019.

6 UP: 2 Die After Inhaling Toxic Gas While Cleaning Septic Tank | NewsClick(Newsclick, 2019).

7 BIS: 11972 (1987): Code of practice for safety precautions to be taken when entering a sewerage system; BIS:18001(2007): Occupational health and safety management systems - Requirements with guidance for use; BIS: 2470-2 (1985): Indian standard code of practice for installation of septic tanks; ASTM C1227-20: Standard Specification for Precast Concrete Septic Tanks; OSHA 3151-12R (2004): Personal Protective Equipment; CPHEEO, 2013: Manual on Sewerage and Sewage Treatment Systems.

8 There is a wide variation in truck sizes and design, since many trucks are not built by companies, but are retrofitted in garages and workshops.

9 For a discussion of variations of containment systems, and resultant implications see Devaraj et al. (2021).
